# A social-ecological model of working from home during COVID-19

**DOI:** 10.1007/s11116-022-10331-7

**Published:** 2023-02-17

**Authors:** Katherine Pawluk De-Toledo, Steve O’Hern, Sjaan Koppel

**Affiliations:** 1 BehaviourWorks Australia, Monash Sustainable Development Institute, Monash University, 3800 Clayton, Australia; 2 Transport Research Centre VERNE, Tampere University, 33014 Tampere, Finland; 3 Monash University Accident Research Centre, Monash University, 3800 Clayton, Australia

**Keywords:** Trip avoidance, Working from home, COVID-19, Telecommuting, Telework, Social-ecological model, Travel demand

## Abstract

Working from Home (WFH) is emerging as a critical measure for reducing transport demand. Indeed, the COVID-19 pandemic has revealed that trip avoidance measures, especially WFH, could help address Sustainable Development Goal 11.2 (creating sustainable transport systems in cities) by decreasing commuter trips by private motor vehicles. This study aimed to explore and identify the attributes that supported WFH during the pandemic and construct a Social-Ecological Model (SEM) of WFH within the context of travel behaviour. We conducted in-depth interviews with 19 stakeholders from Melbourne, Australia and found that WFH during COVID-19 has fundamentally changed commuter travel behaviour. There was a consensus among participants that a hybrid-work model will emerge post-COVID-19 (e.g., working three days in the office and two days at home). We identified 21 attributes that influenced WFH and mapped these attributes across the five traditional SEM levels (intrapersonal, interpersonal, institutional, community and public policy). In addition, we proposed a sixth higher-order level: “global”, to reflect the worldwide phenomena of COVID-19 and computer programs that also supported WFH. We found that WFH attributes were concentrated at the intrapersonal (individual) and institutional (workplace) levels. Indeed, workplaces are key to supporting WFH in the long-term. Whereby, workplace provision of laptops, office equipment, internet connection and flexible work policies enable WFH, and unsupportive organisational cultures and managers are potential barriers to WFH. This SEM of WFH benefits both researchers and practitioners by providing guidance of the key attributes required to sustain WFH behaviours post-COVID-19.

## Highlights

Working from Home (WFH) is a critical measure for reducing transport demand.

21 attributes influenced WFH during COVID-19 in Melbourne, Australia.

Workplaces are key to supporting WFH beyond COVID-19.

Workplace policies, provision of laptops, office equipment and internet connection enable WFH.

Unsupportive organisational cultures and managers are potential barriers to WFH.

Hybrid-work model (i.e., 3 days in office and 2 days at home) is the emerging norm.

## Introduction

Extensive economic, environmental, health and social benefits can be gained from encouraging a shift from private motor vehicle use to more sustainable travel behaviours (United Nations, 2016). The International Energy Agency advocates a triple policy approach of avoid, shift and improve for encouraging sustainable transport (International Energy Agency, 2020). This includes encouraging the avoidance of travel (either partially by reducing trip distances or entirely by encouraging working from home (WFH) and online shopping); shifting travel modes (from private motor vehicle to active travel modes, public transport and carsharing); and making improvements (such as fuel and vehicle efficiencies).

Pre-COVID-19 research investigating travel behaviour change was predominantly focused on mode shift. Which focused on reducing private motor vehicle travel and increasing active and public transport use; notably absent is trip avoidance research (Pawluk De-Toledo et al. 2022). As evidenced by the COVID-19 pandemic, trip avoidance measures (especially WFH), could help address the Sustainable Development Goal (SDG) target 11.2 (creating sustainable transport systems in cities, by decreasing commuter trips, particularly by private motor vehicles (United Nations, 2018).

Although earlier studies examining the environmental benefits of WFH have yielded mixed results. For example, Choo and colleagues (2005) reported modest reductions in vehicle kilometres travelled, whereas later research reported increased non-work trips by people that WFH (Kim et al. 2015). In addition, a pre-COVID-19 systematic review concluded that previous studies generally found a modest reduction in energy use when WFH (Hook et al. 2020) and recent research has estimated reduced energy consumption with WFH, when transportation, office and residential emissions were considered (Navaratnam et al. 2022).

Indeed, WFH is emerging as a significant transport policy lever for reducing travel demand, with the potential to improve the performance of metropolitan transport networks by at least 10–15 per cent, by reducing traffic congestion and public transport crowding (Beck and Hensher 2021a). Recent research estimates that WFH post-COVID19 could decrease peak hour commuter trips by 6 per cent and commuter trips into the central business district by 20 per cent in the city of Melbourne, Australia (Currie et al. 2021). Consequently, if “positive experiences and lessons learnt (about WFH) can be carried forward into a post-pandemic world, it will likely be the largest tool in the transport tool kit to reduce persistent congestion” (Beck and Hensher 2020, p.116–117).

The research field of WFH has a long history stemming from the 1970s, and a growing number of COVID-19 specific studies are emerging. Indeed, previous WFH research is well captured by other researchers (Athanasiadou and Theriou 2021; Bailey and Kurland 2002) and WFH and SDGs (Moglia et al. 2021). However, Beck and colleagues called for further research into WFH within a transport context, to understand the attributes that support trip avoidance as there are concerns that societies post-COVID may adopt less sustainable travel behaviours (Beck et al. 2020). A widely used approach to synthesise attributes and develop behaviour change interventions is the Social-Ecological Model (SEM) (Bronfenbrenner 1977; Mcleroy et al. 1988).

### Social-ecological model (SEM) background

The overarching premise of the SEM is that behaviour is shaped by multiple levels of influence, with interaction across the different levels (Mcleroy et al. 1988; Sallis et al. 2008). Proponents of the SEM argue that a holistic and comprehensive multi-level approach leads to better behaviour change outcomes than individual-level psychosocial interventions, given the multiple levels of influences on behaviour (Sallis et al. 2006).

**Fig. 1:**
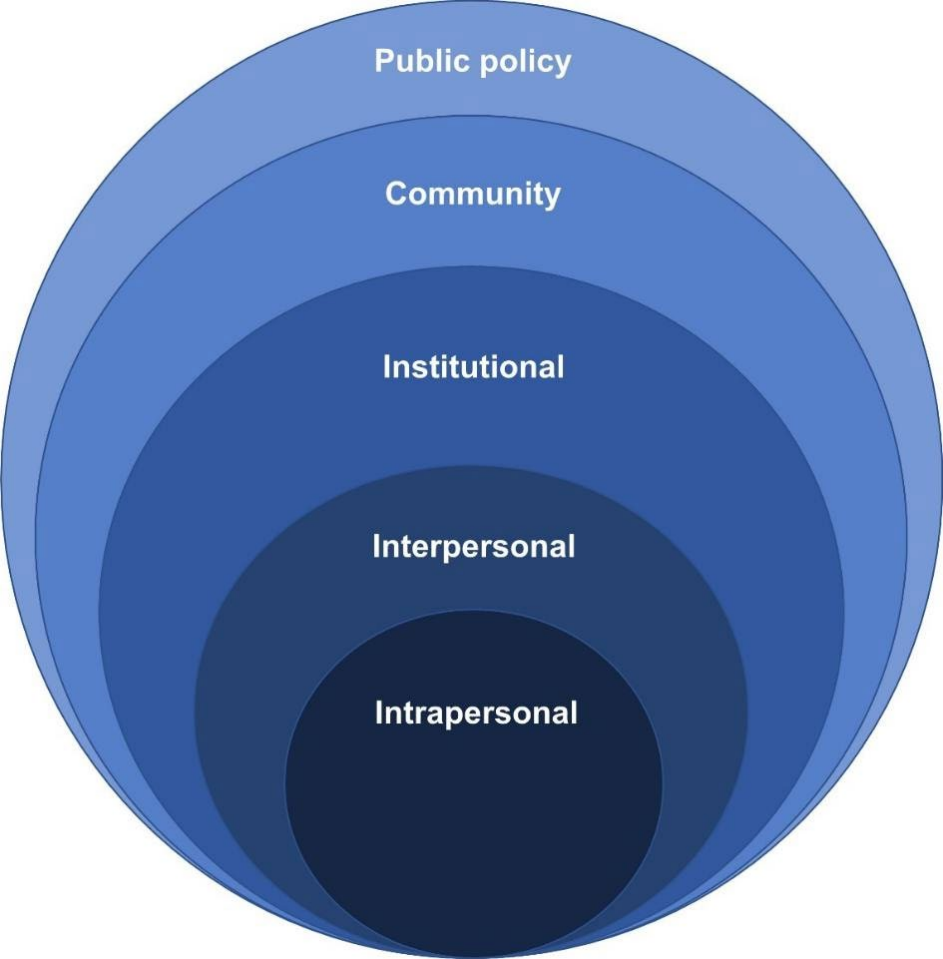
Overview of the SEM proposed by Mcleroy and colleagues (Mcleroy et al. 1988)

The SEM is a systems model, whereby change can occur due to change at any level. Patterned behaviour is the outcome of interest, with behaviour seen to be “affected by, and affecting the social environment” (Mcleroy et al. 1988 p.355). To address the oversimplified, collapsed levels of previous ecological models, Mcleroy and colleagues (1988) defined five levels of analysis of the SEM and proposed the development of behaviour change interventions to target the different levels. The SEM can be visualised as a series of nested levels with the individual in the centre, presented in Fig. 1. Mcleroy and colleagues (1988) defined the five levels in the SEM as follows:

(1) Intrapersonal-level: Individual-level characteristics such as behaviour, knowledge, attitudes, and demographic characteristics.

(2) Interpersonal-level: Formal or informal social networks or support systems (e.g., family, friends, neighbours).

(3) Institutional-level: Organisational settings that exist outside the home (e.g., schools, universities, and workplaces).

(4) Community-level: A group or area based on a range of factors, including geographical, psychological, patterns of social interaction, and political.

(5) Public policy-level: Laws and policies that exist at local, state, and national levels.

While originating in the health behaviour/promotion field, the SEM has been increasingly applied to a broader range of research fields, including travel behaviours (Acheampong and Cugurullo 2019). A 20-year review of the SEM literature published between 1989 and 2008, revealed the main behaviours studied were nutrition, physical activity, smoking, sexual behaviour, and alcohol and substance abuse (Golden and Earp 2012). Over the last decade, since this review, the travel behaviour field has increasingly applied the SEM to specific behaviours (Acheampong and Cugurullo 2019), especially active transport studies (Götschi et al. 2017; Pikora et al. 2003) which have focused on specific trip types: to school (Smith et al. 2020) and the work commute (Aittasalo et al. 2017; Feuillet et al. 2015). In addition, the SEM was used to study adolescent intent to commute by private vehicle or bike as an adult (Sigurdardottir et al. 2013) and office-based physical activity (Van Kasteren et al. 2020).

Travel behaviour studies have also applied the SEM to study particular groups: children’s mobility (Mitra and Manaugh 2019); active travel of college students (Sims et al. 2018); physical activity of older adults (Thornton et al. 2017) and women from socioeconomically disadvantaged areas (Cleland et al. 2020). In addition, several studies have focused on specific travel modes: bicycling (Acheampong and Siiba 2018); walking (Alfonzo 2005); walking for the purposes of walking a dog (Christian et al. 2017; Westgarth et al. 2014); and most recently, autonomous vehicle ownership adoption (Acheampong and Cugurullo 2019).

To the best of our knowledge, the use of the SEM to study WFH within the context of travel behaviour is novel. The SEM is a way of formalising and visualising the plethora of attributes influencing WFH. The SEM of WFH could benefit both researchers and practitioners alike, encouraging the endurance of trip avoidance behaviour post-COVID-19.

### Aims

This study aimed to explore and identify the attributes that supported WFH during COVID-19 and construct a SEM of WFH within a travel behaviour context. To achieve this an exploratory research design was used to identify new attributes that emerged due to the COVID-19 pandemic using the SEM framework.The attributes and model were specific to Melbourne, Australia, which aligns with the recent evolution of SEMs that they should also be location-specific to increase their effectiveness (Feuillet et al. 2015). Melbourne is uniquely positioned to provide valuable lessons to cities globally about WFH due to COVID-19 because it was the most locked-down city in the world and therefore may have crystallised WFH attitudes and behaviours.

The study has been organised as follows: The next section outlines the research methodology. Following this, the results for each level of the SEM are presented and discussed. The final section provides a conclusion, discusses the limitations and proposes future research directions.

## Method

Given the current and ongoing nature of WFH due to COVID-19, a qualitative research method was used to explore and identify the attributes that have influenced the trip avoidance behaviour of WFH during COVID-19. The widespread nature of WFH due to COVID-19, enabled an inductive approach that allowed for new and original WFH attributes to emerge from the study data, rather than by prescribing attributes based on pre-COVID-19 literature. Due to the exploratory nature, this study does not seek to quantitatively measure the relationship of the different attributes on WFH. The Social-Ecological Model (SEM), adapted from social psychology and applied to a transport case study, explores and visualises how different facets of the SEM model contribute to this WFH behavioural outcome.

### Participant sample

The data was collected via in-depth interviews with a diverse group of stakeholders from Melbourne (Australia). Previous transport stakeholder studies have sought to gather perspectives from the diverse range of stakeholders which exist (Ritchie and Lewis 2003; Robson 2011), including public servants from different levels of government, politicians, consultants, NGOs, academics, and representatives from transport providers and vehicle manufacturers (Edge et al. 2020; Moradi and Vagnoni 2018). The three overarching groups used by Steurer and Bonilla (2016) were applied in this study; government, private and civil society. Quota sampling was used to attempt to recruit six participants from each group (Robson 2011), including two women per group as women could raise different issues based on their differing WFH COVID-19 experiences (Feng and Savani 2020; Muric et al. 2021; Yaish et al. 2021; Yavorsky et al. 2021). Researchers knowledge of Melbourne transport stakeholders ensured that key organisations and range of organisations were represented in each group. Interviewing continued until cohort level saturation was reached, defined as no new codes and meanings generated from interview data (Hennink et al. 2017).

### Interview procedure

In-depth interviews were conducted via Zoom videoconference between 14 May and 10 August 2021 and ran for approximately one hour. The interviews were conducted while Melbourne was open and locked-down (Melbourne was in lockdown 34 days, 38.6% of the time interviews were conducted). Participants were encouraged to provide responses about pre-COVID-19 WFH, as well as at specific times during COVID-19 when the city was open, to explore potential differences. All interviews were audio recorded, with participants providing consent for responses to be transcribed. The study received ethics approval from the University Human Research Ethics Committee.

### Interview design

This study analysed the results from a series of semi-structured WFH interview questions (see Appendix 1). All of the responses collected from the WFH questions were included in the analysis. Interview questions were a guide to the semi-structured interview to encourage in-depth discussion about WFH. In addition, responses were included when participants raised WFH in response to introductory questions about the most significant change in travel behaviours since Melbourne’s first lockdown due to COVID-19 in March 2020, and a follow-up question of whether any of these travel behaviour changes will change in the next few years post-COVID-19.

### Data analysis

All the interviews were transcribed and coded using NVivo (version 1.3). During the transcription process, attributes that influenced WFH were highlighted to facilitate coding. However, during the extraction of relevant material from interview transcripts, the entire interview transcript was analysed for coding.

Firstly, all the references to attributes influencing WFH in the interview transcripts were deductively coded to the five levels of the SEM a priori (Saldana 2016). This involved assigning participant data referring to influences on WFH to one of the five levels of the SEM, as outlined by Mcleroy and colleagues (1988), including: intrapersonal, interpersonal, institutional, community and public policy levels. Secondly, the data coded to each SEM levels, were separately synthesised and inductively coded into the emergent attribute themes. Finally, the attributes that emerged at each level were synthesised and visualised into a diagram based on the nested model in Fig. 1.

A large number of information technology (IT) hardware and software comments were made, these were initially coded as technology and then further coded to the different levels of the SEM. This process revealed that there were IT attributes at multiple levels of the SEM including at a global level, which was subsequently added as a six level in the SEM model. Similarly, participant discussions about the impact of COVID-19 on WFH did not fit with the existing levels of the SEM. Given the global experience, references to COVID-19 were coded into the new global level.

## Results and discussion

A total of 19 stakeholder in-depth interviews were conducted. The breakdown of interview participants by stakeholder group, organisation and gender are presented in Table 1.

**Table 1:** Overview of in-depth interview participants

Stakeholder groups		Interview participant (n = 19)	Gender Female (n = 7)
Public sector n = 8	Victorian Public Service	6	2
	Local council	1	1
	Councillor	1	0
Private sector n = 5	Large organisation	3	1
	Transport company	1	1
	Consultant	1	0
Civil Society n = 6	Academia	2	1
	Union	1	0
	NGO/Association	3	1

**Fig. 2:**
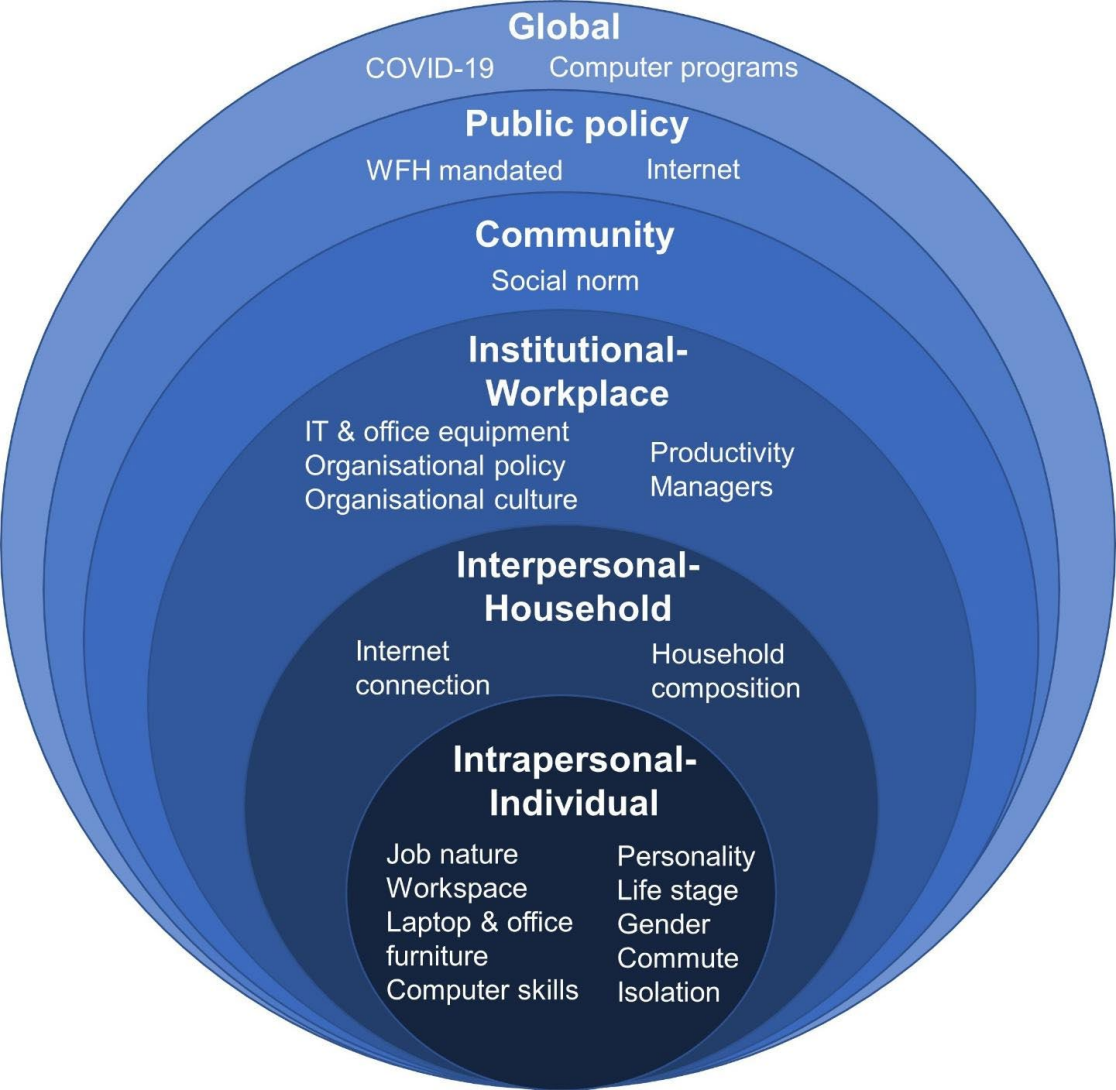
The SEM of WFH for Melbourne, Australia during COVID-19

In total, 21 attributes were extracted from the interview transcript analysis. Based on these attributes, a sixth level was included in the SEM of WFH representing global-level attributes (see Fig. 2). The attributes for each of the levels are presented and discussed in turn with illustrative quotes.

### Intrapersonal-level: individual

The intrapersonal-level relates to individual-level characteristics, including knowledge, attitudes and demographics (Mcleroy et al. 1988). This level had the most identified attributes (n = 9), including: job nature, workspace, laptop and office furniture, computer skills, personality, life stage, gender, commute, and isolation.

#### Job nature

Job nature was seen to significantly influence an individual’s ability to WFH. Participants acknowledged that not all professions could WFH (e.g. manufacturing, construction and medical professions). However, most discussions centred on the paradigm shift that’s occurred in office-based workplaces about what jobs and tasks can be completed from home. Whereby nearly all office jobs, even customer-facing and those handling confidential documents, that were considered office-only jobs pre-COVID-19, can now be done from home.

*“So it (COVID-19) virtually tested all of those barriers about, we can’t work from home, we can’t do those activities. And even some activities that were deemed could only be done in the office, they have now actually moved and transitioned to home.”* -Private sector#5.

#### Workspace

Having the right work environment available at home, ideally a quiet space and most participants specified a home office.

#### Laptop and office furniture

Particularly laptops were deemed to facilitate WFH, as were monitors and ergonomic chairs. While this equipment is essential at an individual level, there’s a crossover with the institutional-level, as office equipment is often provided by workplaces, especially during lockdown (see Institutional level: IT & office equipment).

Discussions highlighted the inconsistent workplace provision of equipment during COVID-19. Some workplaces placed the burden on employees to purchase their own equipment. This could result in unsustainable amounts of equipment when workplaces reopen and employees need both home and office setups, or if equipment purchased for home use is no longer used.

*“…I’ve had to buy new monitors myself, because I had to take my other ones back to work… I had to buy a webcam for this one because there wasn’t one on the monitor. So technology can be a bit of a barrier.”* -Public sector#6.

#### Computer skills

Computer skills were another important attribute that emerged. Participants noted two important aspects. Firstly, technical ability to use the computer programs and secondly, communication skills to effectively participate in online meetings.

*“I think everyone’s IT skills increased and communication skills online got clearer…in the beginning, we were really clumsy in some regard. And people got better with…stopping and listening and taking your turn.”* -Public sector#7.

#### Personality

Participants discussed that an individual’s personality could impact WFH. Participants noted that some staff members switched off their video cameras or did not speak in online meetings, which could reflect someone’s personality. Alternatively, one participant reported that employees with an extroverted personality type could struggle with a lack of interaction and personal connection with WFH.

#### Life stage

Participants reported that, contrary to expectation, younger and older employees were more likely to return to the office when workplaces reopened. In contrast, employees with children were more likely to work from home.

*“So young people come in because they may not have somewhere at home that’s really good to work from. They want to be around people to progress their career…And then the older generation come back in, because they still really think of work in a hierarchical way. So, they think of routine…and all of that…that middle bracket is harder to convince.”* -Private sector#2.

#### Gender

Several participants noted that female employees were impacted differently by WFH. Whereby, women with young children or remote learning experienced increased caring and household workloads during COVID-19. Although, as one participant reflected on her own experience, female employees could benefit from WFH in terms of balancing family responsibilities:

*“…there is a lot of tangible exhaustion out there…and I think it’s more evident in women than in men, because of the imbalance about who does a lot of work at home. And a lot of women just look exhausted.”* -Public sector#3.

#### Commute

Participants reported that the commute time would influence the decision to WFH. Whereby employees that live close to their workplace, commute time is either not a consideration, or they will be more inclined to work in their workplace. Alternatively, employees with longer commute times would be more inclined to WFH. Although, overall participants described the work commute as “time-wasted” and WFH as “time-saving”, which could be used for other work or personal activities.

*“For me the commute time and the amount of time that frees up in my day to do other things, to spend time with the family and friends, to exercise or whatever, that’s such a big gain.” -Public Sector#5*.

*“I suppose the biggest thing is that travel, the commute to work is gone away. Don’t have to commute in the morning, I save half an hour each way…that’s half an hour in the morning of clearing my emails.”* -Public sector#7.

#### Isolation

Feeling isolated was seen to impact WFH negatively. Participants thought people might feel isolated if they lived alone. However, participants acknowledged there had been enormous diversity of experiences due to their household composition (see interpersonal-level: household composition):

*Colleagues said, “Oh I hated lockdown because I lived by myself and I couldn’t move. I just felt so isolated, lonely.” So different for me, I’ve got a wife and three kids, it’s absolute mayhem at home. So it’s a different environment. I don’t think everyone’s in the same shoes.”* -Public sector#8.

#### Intrapersonal-level discussion

The results within this level of the SEM highlight that WFH is influenced by the interplay between three different types of attributes. Including relating to an individual’s work (job nature, having equipment, and skills to perform work), sociodemographic (gender, life stage, and family status) and psychosocial attributes (personality and isolation).

Relating to an individual’s work, job nature influenced the ability to WFH. Several recent studies have found that most jobs cannot be done from home, posing a challenge for encouraging WFH post-COVID-19. Baker (2020) studied the US and found that 75 per cent of jobs cannot be done at home, and Holgersen and colleagues (2021) found that 62 per cent of Norwegian jobs cannot be conducted at home. Indeed, a recent review in the *World Bank Research Observer* found that globally one in five jobs can be done at home, one in three in high-income countries, whereas only one in twenty-six in low-income countries, and highlighted within-country inequalities emerging (Garrote Sanchez et al. 2021).

Other work-related attributes such as having an appropriate workspace also enabled WFH. Indeed, this was found to be one of the main barriers of WFH in a European study (Ipsen et al. 2021), as did a study in India (Nayak and Pandit 2021). Also, having a laptop emerged as essential for WFH. This concurs with an Australia-wide study that found IT software and hardware were key enablers from organisational and employee perspectives (Marzban et al. 2021). However, difficulties accessing data emerged as a challenge in several recent studies (Nayak and Pandit 2021; Nguyen 2021).

Furthermore, we found there is a risk that employees have to purchase their own equipment, which was reinforced by Beck and Hensher (2021), who found that 42 per cent had to pay for the equipment and technology to WFH. While participants in our study discussed that technical ability and online communication skills had improved, there was no mention of receiving training sessions, which Nayak and Pandit (2021) recommend training for new telecommuters.

Looking at sociodemographic attributes, life stage appears to be a mediating factor. For example, having an appropriate workspace is more likely for older Australian workers (Beck and Hensher 2021b). Also, our research found that younger and older people were more likely to come into the office than middle-aged and that middle-aged people with families could be particularly interested in WFH beyond COVID-19. In contrast, recent research found that the middle age group (aged 31–45 years) were less optimistic about WFH post-COVID-19 (Nguyen 2021).

Participants perceived that household workloads had increased during COVID-19, which has been confirmed by other research in Australia (Craig and Churchill 2021) and overseas (Hipp and Bünning 2021; Yerkes et al. 2020). Men were found to increase their involvement in childcare, although women continued to shoulder the burden of housework (Craig and Churchill 2021; Hipp and Bünning 2021). Although, childcare equality was found to wane over time (Hipp and Bünning 2021). Furthermore, more women left paid employment during COVID-19 to perform care duties, mainly if they had young children (Rivera and Castro 2021). Regarding doing their job, a Europe-wide study found that women faced more home office constraints and lacked adequate work tools than their male counterparts (Ipsen et al. 2021). However, despite these gender differences, research has also found that women prefer WFH beyond COVID-19 to men, which also emerged as a potential outcome in our study (Beck and Hensher 2021b; Nguyen 2021).

Indeed, previous research has similarly found that longer commutes were associated with a greater likelihood of WFH post-COVID-19 (Nguyen 2021). Furthermore, research in India found that saving commute travel time was a primary reason for WFH (Nayak and Pandit 2021). Alternatively, our research concurred with Australian research that found isolation was an issue for WFH employees, especially younger people (Marzban et al. 2021). Previous research also similarly found that single people, separated or divorced, or who did not live with a partner experienced more isolation (Groarke et al. 2020; Ortiz-Lozano et al. 2021).

Many of the intrapersonal variables that emerged in our study have been the subject of recent research conducted during COVID-19. While these attributes directly affect the individual, they are also influenced by attributes at multiple levels, especially the interpersonal-and institutional levels, which will be discussed in the following sections.

### Interpersonal-level: household

Interpersonal-level attributes occur at the social and support network level (Mcleroy et al. 1988). The household unit was the only interpersonal-level unit that emerged. Two household-level attributes were found: internet connection and household composition.

#### Internet connection

The internet emerged as a attribute within two of the levels of the SEM. Internet connection quality and stability were noted at the interpersonal level, which varied depending on where individuals lived. In addition, participants discussed it at the policy level (see internet section). However, the implications of poor internet connectivity were felt at the individual level in prohibiting individuals from WFH.

Staff *“actively select to come to work because, they say, “I’m not getting anything done at home, or their internets gone down.” So they go to work”* -Private sector#3.

*“Internet and IT is a big part of it… it’s not half as bad as it could have been, but anyone who has an unstable internet connection, it really makes life difficult at those times.”* -Public sector#4.

#### Household composition

The household composition appears to be an essential determinant for WFH. Participants noted that WFH experiences were diverse, depending on household composition. Several different household examples emerged from the discussions, including: multiple workers simultaneously WFH in a household, the presence of school-aged or young children, living in a share house, and single-person households (also discussed in individual-level: isolation).

*“…that’s quite a different experience from where WFH means you’re sitting at your dining room table surrounded by family members, kitchen noise…”*-Civil society#6.

*“I know for some people that they don’t have that space… I’ve had Zoom calls with people who have been sitting on their balcony.”* -Private sector#4.

Some household circumstances are temporal (e.g. remote learning due to COVID-19). Consequently, participants thought that the flexibility of WFH would be beneficial to employees with school-aged children in the long run. However, several participants highlighted that employees might have complicated relationships at home, for whom going to work is respite, which makes WFH difficult.

*“…you’ve got lots of staff over the years, a lot of them do it really tough at home. There’s a lot of people for whom work is respite…Now those people have had a really tough time during COVID.”* -Public sector#3.

*“…some people obviously don’t want to be home at all, right? Because you’ve got vulnerable people in those categories of people… So going to work is a way in which they, escape or manage whatever they need to manage from.”* -Public sector#6.

#### Interpersonal-level discussion

Two interpersonal-level attributes (internet connection and household composition) strongly impacted WFH experiences. Our results concur with recent research that found WFH experiences during COVID-19 were heterogeneous due to different household compositions (Ortiz-Lozano et al. 2021). Similarly, other studies conducted globally found that household members were a distraction (India: Nayak and Pandit 2021), especially children (Europe: Ipsen et al. 2021; Hanoi: Nguyen 2021; Spain: Ortiz-Lozano et al. 2021). Conversely, workers with children enjoyed being home more during COVID-19 (Ipsen et al. 2021), and isolation was more pronounced for people without children (Ortiz-Lozano et al. 2021). Nevertheless, Nguyen’s study (2021) confirmed an opinion raised in our study, whereby workers with children were found to prefer the idea of WFH post-COVID-19.

We found that home internet connection influenced people’s ability to do their work, which concurs with previous research (Nayak and Pandit 2021). However, we also found that internet connectivity varies widely, depending on home location, which concurs with a UK-wide study (Budnitz and Tranos 2021). While internet connectivity can be improved, potentially through employer provision of work mobile phones with adequate data plans, elements of household composition are much harder to address. While some attributes are temporal (e.g. remote learning), other enduring household circumstances (e.g. people with complicated home lives) make WFH nearly impossible.

### Institutional-level: workplace

In the SEM, this level relates to organisational settings beyond the household (Mcleroy et al. 1988). There was extensive discussion about the institutional-level attributes that related to an employee’s workplace, including: IT and office equipment, organisational policy, organisational culture, productivity and managers.

#### IT & office equipment

As described earlier, participants identified laptops and office equipment (e.g., monitors and ergonomic chairs) as essential items that individuals need to do their jobs. Their workplace generally supplied these, making them intrapersonal and institutional levels (see Intrapersonal-level: laptop and office furniture).

Participants also discussed that WFH was enabled by workplaces’ intranet, internal digital operating systems, and the capacity of these systems. Results revealed that these systems were at different levels of maturity. Some organisations already had systems pre-COVID-19, whereas others had to rapidly invest in technology and develop internal programs.

*“I can’t say that anything that we were doing before COVID we can’t do anymore because of a systems or admin, can still do our job at home, very successfully.”* -Public sector#7.

*“As I’ve said, hardware would be a big one. So getting, a couple of thousand laptops. That’s one. Two is capacity… which is the ability to connect to the network via that laptop.”* -Private sector#2.

#### Organisational policy

Participants noted that workplaces had changed formal employment policy to support WFH beyond lockdowns. For example, the Victorian Public Service, which employs approximately 50,000 employees in Melbourne, announced its default policy of three days working in the office and two days WFH(Victorian Public Sector Commission, 2021). Other participants noted that their workplace had developed similar policies, with a hybrid of three days in the office and two days at home as the dominant model. Participants agreed that organisational policies are in place to support WFH, however organisational culture is a barrier.

*“Policy-wise I think the settings were there, but culturally not so.”* -Private sector#4.

#### Organisational culture

Four organisational culture themes emerged, including:

1. WFH as the ‘norm’ at two different levels of the SEM, at institutional and community levels (see also Community-level: social norm). At an institutional level, participants discussed that WFH was an emerging norm currently undergoing a paradigm shift within organisations.
2. Recognition that it was currently a new and evolving space.
  *“So there’s the individuals, what individuals want to do, there’s what teams want to do, and then there’s what management wants to do. And we’re definitely seeing a lot of flux around all of that. And I think, not just flux, but it’s that learning on the run.”* -Private sector#4.
3. The need for a culture shift within organisations to create genuinely supportive environments for WFH.
  *“It’s (WFH) not an infrastructure program or technology program or a teach them how to use tools program…we’ve got all that, so I’d focus mostly on the culture.”* -Private sector #4.
4. Concerns about maintaining organisational culture and innovating with an absentee workforce, will result in different ways of working together.

*“There’s a timeline that culture will persist because of the relations that are built. As you bring new people into the organisation, you’ve got to sort of invest time in that personal interaction to reinforce the cultural dynamic that the organisation is trying to achieve.”* -Private sector#2.

*“so this concept of workplace experience, as I said, creating those times I bring my whole team in. So you have to be a lot more organised, purposeful…workshopping things, incubating ideas. So I think it will mean that we’ll see different fit out spaces, you won’t go into an organisation and see a sea of desks, you’ll see much larger spaces.”* -Private sector#5.

### Productivity

Productivity discussions focused on two themes: Firstly, WFH productivity and efficiency of online meetings. Secondly, increased trip avoidance by reducing: work commutes (discussed in the interpersonal: commute section); daytime work-related trips; and domestic and international air travel for work purposes.

Firstly, participants highlighted the change in perception about WFH productivity, which could enable WFH to endure beyond COVID-19. There was a consensus that pre-COVID-19, there was a general perception that WFH was less productive, which has now largely been disproven. Participants also noted the improved efficiency of online meetings. Although, participants acknowledged that the increased productivity experienced during COVID-19 might be unsustainable in the long run.

*“I think employers pre-COVID were very much in the view, no you can’t work from home, it’s not possible, there’ll be no productivity, people will stuff around and won’t do any work…I think a lot of employers have been really surprised at the level of productivity.”* -Public sector#8.

*“The meetings tend to go quicker. They tend to start on time, because if you’re not there at the start, then they’ll start without you. Whereas, if you have a face to face meeting, you might hang around five minutes waiting for everybody to arrive, because they’re all having to drive from different places and have got traffic…people don’t want to spend as long meeting online…(people are not) having side conversations.”* -Public sector#6.

The trip avoidance productivity gain was top of mind for some participants. Participants acknowledged that there would be an overall reduction in private motor vehicle volume if work-related daytime trips were reduced. Participants thought online meetings were advantageous for internal meetings where organisations are split across multiple sites within a city or collaboration with interstate offices; and for external stakeholder meetings. Pre-COVID-19 it was commonplace to travel to several offices dispersed across the city during a day, a behaviour that will not continue when workplaces reopen.

*“I can connect to more people virtually than I could ever connect with in the office environment, because never would I bring all my people into that office environment, there’s too many of them, too dispersed.”* -Public sector#2.

*“…everyone comes because it’s just a meeting online, and you can dedicate half an hour…So I’m finding it’s better to connect with external people. And you’re not on the road to get to that external meeting. That’s really important, it’s saving our travel during the day.”* -Public sector#7.

*“That I think has been a game changer I think for a lot of people in actually just realising we can do this meeting online and reduce car trips that way…I think a lot of car trips could be reduced with that simple change.”* Public sector#6.

Pre-COVID-19 Melbourne to Sydney was the busiest flight route in the world. However, participants discussed that online meetings, events, and conferences significantly decrease domestic and international air travel demand. Although, there was an acknowledgement of the limited interaction with online presentations and lack of valuable incidental conversations. Furthermore, there was scepticism about the long-term reduction in business travel or whether reduced work-related travel will result in increased social and recreational travel.

*“Universities are really guilty with the conference circuits. There’s so much travel…And I think we need to rethink that, I’ve heard a lot of talk about international conferences, planning to continue with a hybrid model post-COVID…they’ve recognised that not everybody can afford to travel, time wise or cost wise.”* -Civil society#1.

Reflecting on an event earlier today *“If this was pre-COVID, we’d need to allow an hour and a half, people need to arrive and get their name badge, and say hello to people. And now it’s just going to Zoom in so we can get straight to the discussion.”* -Private sector#1.

*“There’ll certainly be a cost imperative to keep travel down from a business perspective. But I think over time, it will creep back…Because there’s a human element meeting people to do business.”* -Private sector#2.

#### Managers

Participants saw managers as critical for enabling WFH and saw two leadership extremes emerging: supportive progressive versus traditional hierarchical. Also, participants discussed that WFH due to COVID-19 has fundamentally changed work practices, which now needs a change in management style. Consequently, leadership training was recommended to help managers negotiate changes.

*“…perceptions pre-COVID are now no longer, they’ve been proved to be untrue in the large part. The bit that’s different to that is usually the stereotypically the old managing group who may be older and male and white, like their car, and like everybody to be in the office. So that still exists. And it’s still prevalent, but it’s not, doesn’t hold the sway it used to.”* -Private sector#4.

*“For some people, I think they’ve struggled with the conflict between the amount of autonomy that they’ve had a home, and then going back into the office and finding that the expectations of their coordinators…that they’ve gone sort of backwards,…they’ve been allowed to be autonomous at home, and then suddenly they’re back in the office and being dictated to.”* -Public sector#6.

*“…if you’re someone who looked at someone sitting at their desk from eight to five, and that’s how you evaluate their work effort during the day. And that will be quite tricky for you. And there are still those leaders out there. And they’ve had to bring them on a journey and for an educational piece.”* -Private sector#5.

#### Institutional-level discussion

Institutional-level results highlighted five workplace attributes that influence WFH: IT and office equipment, organisational policy, organisational culture, perception of WFH productivity and managers.

The provision of IT and equipment (especially laptops) by employers to staff was a key enabler for people to do their work (Also discussed Intrapersonal section: Laptop and office furniture). However, participants raised that employer provision was not universal, indeed the burden of purchasing equipment was sometimes placed on the employee. This concurs with a recent Spanish study that found ambiguous resources (e.g. internet, computer, chair) provided by employers (Ortiz-Lozano et al. 2021), and in the Australian context, 42 per cent of those surveyed had to pay for their own equipment and technology to WFH (Beck and Hensher 2021b).

From an organisational perspective, our results revealed different levels of IT preparedness for widespread WFH in Melbourne pre-COVID-19. This concurs with previous Australia-wide research that found IT infrastructure (software and hardware) as a critical WFH enabler from both employee and organisational perspectives and also one of the top five challenges for organisations (Marzban et al. 2021).

In our study, participants perceived that a hybrid model of work would be commonplace in Melbourne in the future, enabled by formalised employment policies. This concurs with an Australia-wide study that found flexible working policies were the primary enabler for WFH by 72 per cent of employee respondents (Marzban et al. 2021). However, our participants acknowledged that while employment policies are an essential first step, they believed they need to be reinforced by supportive organisational cultures. Participants discussed that it appears that WFH was becoming a norm in organisations. However, they acknowledged that it is an evolving space, and if workplaces fail to make the paradigm shift, it could pose a barrier to WFH enduring.

Our study also revealed that, since COVID-19 there had been a dramatic shift in workplace’s perception of WFH productivity and associated trip avoidance, which concurs with survey results in the US (Barrero et al. 2021; Bick et al. 2021). Pre-COVID-19 WFH was generally seen to be an unproductive way of working that has now largely been dismissed, which participants saw as a critical evolution for supporting WFH enduring. This perception was also confirmed in Australia-wide research from employee and employer perspectives (Beck and Hensher 2021b). However, it contrasts with another Australian study that found productivity is one of the main challenges for organisations with WFH (Marzban et al. 2021). Also, a global survey found that most managers are still unsure or sceptical about the productivity of people WFH relative to in the office (Parker et al. 2020).

The final institutional-level attribute that emerged in our study was managers’ critical role in enabling WFH, with divergent styles emerging (supportive progressive versus traditional hierarchical). Beck and Hensher (2021b) also found divergent experiences between Australia’s metropolitan and regional, with metropolitan areas more supportive than regional areas. They also found more positive perceptions of the average number of working days were held by employers/managers than employees (employers/managers: 2.4 days; employees: 1.7 days), although for both groups, the average number of days decreased between survey waves (Beck and Hensher 2021b). Conversely, a recent study found that unreasonable expectations and close monitoring of employees led to an ‘always on’ culture, work-life imbalance, and stress (Parker et al. 2020). Consequently, as with our results, these researchers also recommend that managers benefit from training that will enhance their management skills and improve employee wellbeing (Parker et al. 2020).

### Community-level

The SEM takes a broad definition of community, relating to a group or area unified by various features, including geography, social interaction and politics (Mcleroy et al. 1988). In this study, participants identified one community-level attribute: WFH is a social norm.

#### Social norm

Participants noted that, since COVID-19, WFH has become a social norm. They mainly discussed the contrasting WFH experiences between pre- and during COVID-19. There was a consensus view that pre-COVID-19 employees needed to have a reason to WFH, essentially if they were sick, expecting a delivery or tradesperson, or had a specific work task to do (i.e., writing that required concentration). However, participants now felt that there is no longer a need to justify WFH. Several participants highlighted that the social norm of WFH could be unique to Melbourne and possibly Sydney, where employees were exposed to more extended periods of WFH. Whereas cities with shorter periods of mandated WFH will have less WFH in the long run.

(Pre-COVID-19) *“There was a reason to be at home, you always felt like you needed a reason, which I think is a thing that COVID has changed…now it’s I’m working from home, because this is what I’m doing today.”* -Private sector#1.

*“I guess you’ll see that even on a city level, where cities that haven’t experienced lockdown, the same as, say Sydney and Melbourne, will maybe have a really different approach to work from home policies that will last for decades.”*-Private sector#1.

#### Community-level discussion

This study found that WFH is now a social norm at two different levels of the SEM, as discussed earlier at an institutional workplace level and more generally at a city-wide level. Our findings revealed that the length of exposure to WFH positively influences the endurance of WFH. While our results cannot be generalised beyond Melbourne, Beck and Hensher (2021b) suggest a similar correlation in their Australia-wide study. Similarly, other recent research found that WFH endurance is influenced by prolonged exposure to WFH during COVID-19 (van Zoonen et al. 2021) or if people had WFH pre-COVID-19 (Nguyen 2021). Indeed, improving our understanding of the duration of exposure to WFH required to result in WFH enduring post-intervention would be valuable for creating more effective WFH interventions.

### Public policy-level

The public policy level relates to the laws and public policies that influence behaviour (Mcleroy et al. 1988). Participants raised two government policies that influenced WFH: (1) that the government mandated WFH, and (2) the internet network.

#### WFH mandated

State governments in Australia enforced state and city level policies of lockdowns and mandatory WFH to curtail the spread of COVID-19. Several participants referred to the mandating of WFH as a critical enabler of WFH:

*“…let’s be really honest, it’s necessity that probably really facilitated it* (WFH).*”* -Civil society#2.

#### Internet

In Australia, a national government policy governs the rollout of residential broadband internet across the country called the National Broadband Network (NBN) (Australian Government, 2011). Many participants raised the importance of the internet in enabling WFH. However, several participants expressed unstable internet as a challenge for WFH (also see Interpersonal section: Internet connection):

*“So I think, sort of internet, broadband infrastructure is a big part of it. Yeah, I think my impression is that the further away from the city you get, the less reliable internet connections are… you can’t WFH unless you have a reliable connection.”* -Private sector#4.

#### Public policy-level discussion

Two policy-level attributes (mandated WFH and the internet) emerged as WFH enablers. They are interrelated with other SEM level attributes, whereby other level attributes appear to stem from the impact of these public policies. Firstly, the combination of the government mandating WFH and the long duration of mandated WFH in Melbourne has influenced the emergence of the community-level attribute of WFH as a social norm (see Community-level: social norm). However, government-mandated lockdowns and WFH were enabled by governments enacting emergency powers to respond to the COVID-19 pandemic, which will not be available to governments in the long term. Instead, governments have incentive mechanisms available to encourage WFH. For example, there are already taxation mechanisms in place which could be improved. Businesses and individuals that work from home can claim expenses relating to depreciation of office furniture, heating, cooling, lighting, phone, internet and stationery (Australian Taxation Office, 2020). Similarly, other researchers have recently suggested that governments, particularly transport authorities, work with industry to create incentives for businesses to encourage further WFH (Beck and Hensher 2021b).

Also, the internet emerged at two levels of the SEM. At the public policy level where the federal government has nationally managed it. Also, at the interpersonal level, the internet connection quality varied depending on home location. The internet connection quality was discussed as a barrier to WFH for individuals, resulting in the inability to participate in meetings and perform work tasks. As we suggested in the interpersonal section, workplace involvement could be encouraged to provide work mobile phones with adequate internet data plans for employees to WFH. Individuals are impacted while conducting work activities, so attention should focus on how to address internet connection through the most SEM impacted levels- individual and institutional.

### Global-level

In this study, individual, household, workplace, city, state and national level attributes were all found to influence WFH. In addition, two higher-order attributes emerged that fell beyond the scope of the five-level SEM framework: COVID-19 and Computer programs. Given the overriding nature of these attributes in creating the unique WFH context, we felt they warranted their own level. Consequently, an additional sixth higher level to the SEM was created, called global-level, reflecting the worldwide scale of these attributes (see Fig. 2).

#### COVID-19

WFH arose in direct response to, and amidst, the backdrop of a global pandemic. Given the worldwide nature of COVID-19, it is a global level attribute and it has also impacted other SEM levels. Participants noted that COVID-19 was the reason WFH was mandated (see Public policy level: WFH mandated). Locational differences (e.g., decision to mandate WFH and lockdown cities) was not determined at a global level but at a city or country level. Also, COVID-19 created a stressful environment for undertaking WFH. For example, multiple family members WFH simultaneously in the one house or juggling the requirements of WFH and remote learning for children (see Interpersonal: Household composition). Participants acknowledged the enormity of the impacts and the potential lasting legacy of COVID-19 on WFH.

*“I think what has happened is COVID has given us a change in the way we work on steroids, because it had to happen. So you had to adapt quickly. So think if we hadn’t have had COVID, this would have taken ages, whether it would have ever happened.”* -Private sector#5.

*“And we’ve had this major disruption and it is shaking the normal and we have the opportunity to (reset)…WFH will be part of that reset…as horrible as COVID has been, what a wonderful opportunity it is for us to reset and rethink the way we want to work and live in society.”* -Private sector#3.

#### Computer programs

Participants discussed how computer programs such as Zoom, Microsoft Teams, Google Docs and Slack had facilitated WFH. International companies designed these computer programs and made the software accessible freely or inexpensively. The programs are used at individual, team, company, cross-institution, national and international levels, consequently creating global communication channels.

These programs are distinguishable from institutional-level software, which have been tailormade to the specifications of that organisation (see Institutional-level section: IT and office equipment). Instead, these computer programs are global, with institutions and individuals adopting them to facilitate work practices. In particular, participants thought video conferencing facilitated WFH. Even when offices partially reopened in Melbourne, meetings were all accompanied by a video conference, which participants reflected was not commonplace before COVID-19. However, several participants commented on experiencing online meeting fatigue. Another downside was half-hour online meetings scheduled, resolvable by quick phone calls. Some organisations overcame this by using other available software such as Slack and collaborating on software such as Google Docs, shared drives and Jira boards.

*“It’s worked extraordinarily…far better than anybody ever would have dreamed…board meetings for four or five hours on quite complex things on Zoom. And yes it’s exhausting. But it’s quite doable. And honestly, if you told me that a year ago, I would have thought, well no that won’t work, I have to be in the room, I need to make eye contact.”* -Public sector#3.

Video conferencing *“20 years ago, was where you have to go to a specific building and sit in a room and pay $100 for someone to connect you up to someone else. That’s now changed so that anyone can just use their own internet connection, put a piece of free software on their computer, and just point, click and go.”* -Civil society#6.

*“Slack was particularly important for those like quick, incidental conversations, or trying to replicate those. And the collaborative nature of Google Docs as well.”* -Private sector#1.

#### Global-level discussion

Two global-level attributes emerged in this study: COVID-19 and computer programs. Participants perceived that both attributes were crucial facilitators of WFH. COVID-19 was the impetus for mandating WFH, the significance of which cannot be understated given that pre-COVID-19 WFH was a niche behaviour (Beck and Hensher 2021b). Now due to the COVID-19 WFH experience, participants believe the nature of working in Melbourne has changed forever. This concurs with other Australian research, which found that 56 per cent of organisations in their study saw increased WFH continuing post-COVID-19 (Marzban et al. 2021). However, COVID-19 is a temporal attribute in the long-term, as cities emerge from lockdowns and mandatory WFH ceases.

The other global attribute, computer programs are closely aligned to the other software attribute that emerged at the institutional level of this study (see IT & office equipment). Our results found that computer programs were a key enabler of WFH. Similarly, Schwarz and colleagues (2020) study of academia found that computer programs successfully enabled a wide range of academic activities, from delivering classes to running global conferences. As with our research, they concluded that a hybrid model of working would continue (Schwarz et al. 2020).

## Conclusion

To our knowledge, this is the first time WFH as a trip avoidance measure has been analysed through a SEM lens. Prior to COVID-19, WFH was seen as a niche behaviour (Beck and Hensher 2021b), however, the COVID-19 pandemic has revealed that WFH is possibly the most important transport lever for addressing traffic congestion (Beck and Hensher 2020). Furthermore, WFH could have a positive impact on achieving the Sustainable Development Goal 11.2 (creating sustainable transport systems in cities) by decreasing work-related trips (United Nations, 2018). However, despite the enormous potential of WFH as a trip avoidance measure, there has been a lack of research to date (Pawluk De-Toledo et al. 2022). This study aimed to address this research gap.

Our research corroborates findings of other recent research that COVID-19 has fundamentally challenged work practices and entrenched preconceptions and social norms about where work can effectively be completed (Beck and Hensher 2021b). There was a consensus among participants in our study that a hybrid work model will emerge in Melbourne post-COVID-19. Specifically, working three days in the office and two days at home appears to be the emerging norm, which at a city-wide scale, would have a significant impact on reducing commute trips. It is estimated that for Melbourne, WFH post-COVID19 could decrease peak hour commute trips by 6 per cent and commute trips into the central business district by 20 per cent (Currie et al. 2021), however potential reductions in previous commuter trips could release latent demand. Consequently, as WFH emerges as a potentially significant influence on urban transport systems, we recommend that city transport visions be revisited to incorporate this unexpected but important transport lever.

WFH was mandated in response to a public health crisis, now it is an opportune time to take a more considered and strategic approach to encourage WFH as a trip avoidance measure in the long-term. This study contributes to supporting this evolution. Our study identified 21 attributes influencing WFH during COVID-19 in Melbourne, Australia. These attributes were spread across the five different levels of the SEM, in addition we proposed the addition of a sixth higher level: global, to reflect the worldwide phenomena of COVID-19 and computer programs that also impacted WFH.

Our multi-level results concur with the underlying premise of the SEM that behaviours occur as the result of multiple and interrelated social-ecological levels of influence (Mcleroy et al. 1988; Sallis et al. 2008). Our study identified attributes at all SEM levels, although some attributes are temporary (e.g., COVID-19, WFH mandated). Overall, attributes were concentrated at the intrapersonal (individual) and institutional (workplace) levels, consequently they are important for encouraging the new normal of WFH. Indeed, workplaces emerged as key to encouraging WFH. Five attributes were identified at the institutional workplace level (IT and office equipment, organisational policy, organisational culture, productivity and managers). In addition, laptops and office equipment were an attribute across levels. Whereby, organisations should be encouraged to support individuals WFH, by equipping them with laptops and ensuring that the burden does not fall on employees. Furthermore, given the interrelated nature of the levels of the SEM, several attributes located at other levels are best addressed through workplaces. For example, internet emerged as an attribute at both interpersonal (household) and public policy levels, however potentially best improved by workplace provision of mobile phones with internet data plans for employees to use while WFH.

Consequently, this study highlights that through the SEM, understanding all the determinants of a behaviour, that solutions to barriers can lie at other levels. Indeed, the units of analysis that emerged in this study (e.g. individual, household and workplace) are applicable to other transport studies that want to investigate the determinants of a behaviour (e.g. bicycle or public transport commutes).

### Recommendations to workplaces and governments

Drawing on the attributes that can be addressed by workplaces, we have developed recommendations for workplaces and governments wishing to support WFH. We recommend four elements: establishing policy, encouraging WFH friendly culture, supplying equipment and delivering training. Workplaces need to review and develop WFH employment policies and company culture policies, and reinforce these measures by proactively encouraging these values within the organisation. Workplaces also need to provide equipment (e.g. laptops and mobile phones with data plans) and deliver training to staff (e.g. computer program and online communication) and managers so they are skilled to support WFH.

Governments could support WFH in three ways: provide support to workplaces, provide tax rebates to employees, and lead by example. There was concern among participants about smaller companies having the resources to support WFH. Consequently, governments could play a vital supportive role by providing informational and training materials to workplaces. Including, providing examples of flexible workplace policies and case studies of successful WFH organisations. Offering online training for managers and occupational health and safety to ensure safe workstation setup. Governments could also provide employees with tax rebates for setting up and running home workstations and on personal telephone and internet bills. Finally, governments should also lead by example. They are large employers, so they should be at the forefront of implementing the workplace WFH recommendations.

### Limitations and future research recommendations

Workplaces are central for the new normal of WFH to endure. Consequently, further research from an organisational perspective is needed. For example, organisational case studies could be conducted to improve the understanding of barriers (e.g., organisational culture and managers) and facilitators of WFH.

There are several limitations to this study. Firstly, this study investigated WFH from a stakeholder perspective, although given the widespread nature of the experience, the participants were also subjected to WFH. However, further insight would be gained from exploring WFH from a broader employee perspective. Also, saturation was reached at the cohort level, which overlooks differences between groups. Indeed, future research could examine inter-group similarities and differences.

This study identified a wide range of attributes that influenced WFH. Given the volume of research emerging on WFH, a valuable follow-up study would be to map the WFH literature against the SEM model developed in this study. Also, many individual attributes (such as gender, life stage, and length of WFH exposure) warrant further exploration. Furthermore, infection fear has been identified as a motivator for WFH, which did not emerge in this study, perhaps because workplaces never entirely reopened at the time of the interviews. This issue should be addressed in future research now that workplaces have reopened in Melbourne.

Furthermore, this study was qualitative and did not statistically examine the relative importance of the different attributes. Indeed, a follow-up study, such as an extensive survey, is the critical next step to identify priority attributes. Also, longitudinal research would also improve our understanding of both the endurance of WFH and the relative influence of each of the attributes at different points in time.

This research also has a temporal limitation, interviews were conducted in mid-2021 when Melbourne was open and during lockdowns. Since then, Melbourne has been crowned the most locked-down city in the world (Boaz 2021). The results could have been different if interviews had been conducted after the latest lengthy lockdown. Furthermore, our study focused on Melbourne, Australia and cannot be generalised to other cities in Australia or globally. Indeed, replicating this study in other cities and countries would further improve our understanding of WFH, its barriers and facilitators.

Our study revealed that work-related trip avoidance could reduce travel demand in three ways, by reducing: (1) am and pm work commutes; (2) daytime work-related trips; and (3) domestic and international air travel for work purposes. Encouraging trip avoidance for each of these three travel types could radically improve the sustainability of urban transport networks and air travel, and as such, each deserves further investigation.

We believe that the SEM has been a valuable approach for analysing the attributes that shape WFH. Whereby encouraging WFH as a trip avoidance measure beyond COVID-19 will require a combination of attributes to be addressed across different levels of the SEM. This SEM of WFH benefits both researchers and practitioners by outlining the critical attributes required to sustain WFH behaviours post-COVID-19.

## Appendix 1 – WFH interview questions.

1. **Participant’s WFH experience**.

- Did you WFH prior to COVID-19?

If yes: How often?

Why?

b) Do you currently WFH?

If yes: How often?

Why?

c) Do you intend to WFH in the future?

If yes: How often?

Why?

**Participant’s general thoughts on WFH**.

2. Since COVIDSafe Summer restrictions were introduced mid-January 2021 (private sector workplaces reopened at 50% capacity and public sector 25%)- what do you think WFH practices are currently in Metropolitan Melbourne?
3. Do you think WFH experiences currently vary across different areas of the city, specifically Melbourne City & Monash NEIC?

If yes, Why?

4. How do you think workplaces currently view WFH? How does your workplace view WFH?
5. How do you think policy makers and government in Victoria currently view WFH?
6. What do you think are the main **benefits** of WFH?
7. What do you think are the main **negative consequences** of WFH?
8. What do you see as the main **barriers** to WFH?
9. What do you see as the main **facilitators** to WFH?
10. How enduring do you think WFH will be in the future in Metropolitan Melbourne?
11. In the short-term (until 2030) how do you think WFH experiences will differ in different areas of the city, specifically Melbourne City & Monash NEIC?
12. What actions would you recommend to encourage WFH? Eg What policy levers (carrots/sticks) would you use to encourage the uptake of WFH?
